# Supplementary material for: Outcomes of Community-Based Systematic Screening of Household Contacts of Patients with Multidrug-Resistant Tuberculosis in Myanmar
Source: Trop Med Infect Dis. 2019 Dec 25;5(1):2. doi: 10.3390/tropicalmed5010002 (PMC7157714; doi:10.3390/tropicalmed5010002)
Supplement: Supplementary file 1 [file tropicalmed-05-00002-s001.pdf]

Annex S1: Household contact screening register used in the community-based MDR-TB Care Project in 33 townships

Reg. No.	Reg. Date *dd/mm/yy*	Name		Address & Phone No:				Symptom	Case found at	Index case Reg No.	Risk Factor	HIV Date	Sputum Date	Antibiotic trial
Township	Referral type	Age	Sex	Refer from				*Select more than one*		Relationship	*Select more than one*	Result	Result	
								☐ Cough ☐ Fever ☐ Weight Loss<br>☐ Blood Stained Sputum ☐ Night Sweat<br>☐ Lymph Node Enlargement<br>☐ Other : ____________	☐ HE<br>☐ TB CT<br>☐ MDRTB CT<br>☐ Community		☐ HIV<br>☐ DM<br>☐ Past History of TB<br>☐ Under 5<br>☐ TB contact<br>☐ MDRTB contact	☐ Not Done<br>☐ Done<br>Date ……/……/……	☐ Not Done<br>☐ Done<br>Date …../……/…..	☐ YES
	☐ Self<br>☐ Sputum		☐ M<br>☐ F	☐<br>Vol	☐<br>GP	☐<br>Phar	☐<br>Self			☐ Household member<br>☐ Non-household member		☐ Negative<br>☐ Positive<br>Code……………	☐ Negative<br>☐ Positive	☐ NO
								☐ Cough ☐ Fever ☐ Weight Loss<br>☐ Blood Stained Sputum ☐ Night Sweat<br>☐ Lymph Node Enlargement<br>☐ Other : ____________	☐ HE<br>☐ TB CT<br>☐ MDRTB CT<br>☐ Community		☐ HIV<br>☐ DM<br>☐ Past History of TB<br>☐ Under 5<br>☐ TB contact<br>☐ MDRTB contact	☐ Not Done<br>☐ Done<br>Date ……/……/……	☐ Not Done<br>☐ Done<br>Date …../……/…..	☐ YES
	☐ Self<br>☐ Sputum		☐ M<br>☐ F	☐<br>Vol	☐<br>GP	☐<br>Phar	☐<br>Self			☐ Household member<br>☐ Non-household member		☐ Negative<br>☐ Positive<br>Code……………	☐ Negative<br>☐ Positive	☐ NO
								☐ Cough ☐ Fever ☐ Weight Loss<br>☐ Blood Stained Sputum ☐ Night Sweat<br>☐ Lymph Node Enlargement<br>☐ Other : ____________	☐ HE<br>☐ TB CT<br>☐ MDRTB CT<br>☐ Community		☐ HIV<br>☐ DM<br>☐ Past History of TB<br>☐ Under 5<br>☐ TB contact<br>☐ MDRTB contact	☐ Not Done<br>☐ Done<br>Date ……/……/……	☐ Not Done<br>☐ Done<br>Date …../……/…..	☐ YES
	☐ Self<br>☐ Sputum		☐ M<br>☐ F	☐<br>Vol	☐<br>GP	☐<br>Phar	☐<br>Self			☐ Household member<br>☐ Non-household member		☐ Negative<br>☐ Positive<br>Code……………	☐ Negative<br>☐ Positive	☐ NO
								☐ Cough ☐ Fever ☐ Weight Loss<br>☐ Blood Stained Sputum ☐ Night Sweat<br>☐ Lymph Node Enlargement<br>☐ Other : ____________	☐ HE<br>☐ TB CT<br>☐ MDRTB CT<br>☐ Community		☐ HIV<br>☐ DM<br>☐ Past History of TB<br>☐ Under 5<br>☐ TB contact<br>☐ MDRTB contact	☐ Not Done<br>☐ Done<br>Date ……/……/……	☐ Not Done<br>☐ Done<br>Date …../……/…..	☐ YES
	☐ Self<br>☐ Sputum		☐ M<br>☐ F	☐<br>Vol	☐<br>GP	☐<br>Phar	☐<br>Self			☐ Household member<br>☐ Non-household member		☐ Negative<br>☐ Positive<br>Code……………	☐ Negative<br>☐ Positive	☐ NO
								☐ Cough ☐ Fever ☐ Weight Loss<br>☐ Blood Stained Sputum ☐ Night Sweat<br>☐ Lymph Node Enlargement<br>☐ Other : ____________	☐ HE<br>☐ TB CT<br>☐ MDRTB CT<br>☐ Community		☐ HIV<br>☐ DM<br>☐ Past History of TB<br>☐ Under 5<br>☐ TB contact<br>☐ MDRTB contact	☐ Not Done<br>☐ Done<br>Date ……/……/……	☐ Not Done<br>☐ Done<br>Date …../……/…..	☐ YES
	☐ Self<br>☐ Sputum		☐ M<br>☐ F	☐<br>Vol	☐<br>GP	☐<br>Phar	☐<br>Self			☐ Household member<br>☐ Non-household member		☐ Negative<br>☐ Positive<br>Code……………	☐ Negative<br>☐ Positive	☐ NO

TST	CXR Date	GeneXpert Date	FNAC Date	Culture Date	DST Date	Conclusion/Treatment received	Other/comment
Result	Result	Result	Result	Result	Result		Result
□ Not Done<br>□ Done<br>Date …../……/……	□ Not Done<br>□ Done<br>Date ……/……/……	□ Not Done<br>□ Done<br>Date ……/……/……	□ Not Done<br>□ Done<br>Date ……/……/…..	□ Not Done<br>□ Done<br>Date ……/……/……	□ Not Done<br>□ Done<br>Date ……/……/……	□ TB Code_______________<br>□ MDRTB Code_______________<br>□ IPT Code_______________<br>□ No TB<br>□ No IPT	
Size mm …………	□ Normal<br>□ Abnormal	□ MTB Not Detected<br>□ MTB Detected<br>□ Rif Resistance	□ Negative<br>□ Positive	□ Negative<br>□ Positive	□ Resistant H □ Resistant R<br>□ Resistant Z □ Resistant E<br>□ Resistant S		
□ Not Done<br>□ Done<br>Date …../……/……	□ Not Done<br>□ Done<br>Date ……/……/……	□ Not Done<br>□ Done<br>Date ……/……/……	□ Not Done<br>□ Done<br>Date ……/……/…..	□ Not Done<br>□ Done<br>Date ……/……/……	□ Not Done<br>□ Done<br>Date ……/……/……	□ TB Code_______________<br>□ MDRTB Code_______________<br>□ IPT Code_______________<br>□ No TB<br>□ No IPT	
Size mm …………	□ Normal<br>□ Abnormal	□ MTB Not Detected<br>□ MTB Detected<br>□ Rif Resistance	□ Negative<br>□ Positive	□ Negative<br>□ Positive	□ Resistant H □ Resistant R<br>□ Resistant Z □ Resistant E<br>□ Resistant S		
□ Not Done<br>□ Done<br>Date …../……/……	□ Not Done<br>□ Done<br>Date ……/……/……	□ Not Done<br>□ Done<br>Date ……/……/……	□ Not Done<br>□ Done<br>Date ……/……/…..	□ Not Done<br>□ Done<br>Date ……/……/……	□ Not Done<br>□ Done<br>Date ……/……/……	□ TB Code_______________<br>□ MDRTB Code_______________<br>□ IPT Code_______________<br>□ No TB<br>□ No IPT	
Size mm …………	□ Normal<br>□ Abnormal	□ MTB Not Detected<br>□ MTB Detected<br>□ Rif Resistance	□ Negative<br>□ Positive	□ Negative<br>□ Positive	□ Resistant H □ Resistant R<br>□ Resistant Z □ Resistant E<br>□ Resistant S		
□ Not Done<br>□ Done<br>Date …../……/……	□ Not Done<br>□ Done<br>Date ……/……/……	□ Not Done<br>□ Done<br>Date ……/……/……	□ Not Done<br>□ Done<br>Date ……/……/…..	□ Not Done<br>□ Done<br>Date ……/……/……	□ Not Done<br>□ Done<br>Date ……/……/……	□ TB Code_______________<br>□ MDRTB Code_______________<br>□ IPT Code_______________<br>□ No TB<br>□ No IPT	
Size mm …………	□ Normal<br>□ Abnormal	□ MTB Not Detected<br>□ MTB Detected<br>□ Rif Resistance	□ Negative<br>□ Positive	□ Negative<br>□ Positive	□ Resistant H □ Resistant R<br>□ Resistant Z □ Resistant E<br>□ Resistant S		
□ Not Done<br>□ Done<br>Date …../……/……	□ Not Done<br>□ Done<br>Date ……/……/……	□ Not Done<br>□ Done<br>Date ……/……/……	□ Not Done<br>□ Done<br>Date ……/……/…..	□ Not Done<br>□ Done<br>Date ……/……/……	□ Not Done<br>□ Done<br>Date ……/……/……	□ TB Code_______________<br>□ MDRTB Code_______________<br>□ IPT Code_______________<br>□ No TB<br>□ No IPT	
Size mm …………	□ Normal<br>□ Abnormal	□ MTB Not Detected<br>□ MTB Detected<br>□ Rif Resistance	□ Negative<br>□ Positive	□ Negative<br>□ Positive	□ Resistant H □ Resistant R<br>□ Resistant Z □ Resistant E<br>□ Resistant S		
